# Supplementary material for: Genetics and Sport Injuries: New Perspectives for Athletic Excellence in an Italian Court of Rugby Union Players
Source: Genes (Basel). 2022 Jun 1;13(6):995. doi: 10.3390/genes13060995 (PMC9223017; doi:10.3390/genes13060995)
Supplement: Supplementary file 1 [file genes-13-00995-s001.zip › genes-1711640-supplementary.pdf]

Supplementary tables: Table S1. DNA genotyping and injuries collection in the athletes' group.

ID	*GENOTYPE*				*INJURIES*			
	ACTN3	*ACE*	*MCT1*	*COL1A1*	*Muscles*	*Tendons*	*Bones*	*Total*
**001**	CC	DD	AT	TT	2	3	4	9
**002**	CT	ID	AT	GG	1	6	0	7
**003**	TT	ID	AT	GT	1	4	6	11
**004**	TT	II	AT	GG	2	3	7	12
**005**	TT	ID	AA	TT	3	2	4	9
**006**	TT	ID	AA	TT	1	1	7	9
**007**	TT	DD	AT	GT	1	2	5	8
**008**	CT	DD	AA	GG	0	2	3	5
**009**	CT	ID	AA	GT	2	1	5	8
**010**	CT	DD	AT	GT	2	1	4	7
**011**	CT	DD	AT	GT	1	2	9	12
**012**	TT	ID	AA	GG	1	5	2	8
**013**	CT	II	TT	GG	1	1	5	7
**014**	CT	ID	AT	GG	1	0	3	4
**015**	CT	II	AT	GT	0	4	1	5
**016**	CT	ID	AA	GT	1	2	11	14
**017**	CC	ID	AA	GT	2	0	3	5
**018**	CC	II	AT	GG	0	0	2	2
**019**	TT	ID	AA	GG	0	1	7	8
**020**	CC	ID	TT	GG	2	3	4	9
**021**	CC	ID	AA	GG	1	1	6	8
**022**	CT	II	AA	GT	6	3	14	23
**023**	CT	II	TT	GT	0	4	3	7
**024**	TT	II	AT	GG	0	4	2	6
**025**	CT	II	AT	GT	0	1	2	3
**026**	CT	DD	AT	GT	2	0	4	6
**027**	CT	ID	AA	GG	1	1	2	4
**028**	CC	ID	AT	GG	2	3	6	11
**029**	CC	ID	AA	GG	2	9	14	25
**030**	CT	DD	AT	GT	2	0	1	3
**031**	CT	II	AA	GG	2	3	6	11
**032**	CT	DD	AT	GG	1	2	7	10
**033**	CT	ID	AT	GG	0	2	12	14
**034**	CC	ID	AA	GG	2	4	7	13
**035**	CT	ID	AT	GT	2	2	10	14
**036**	CC	ID	AT	GG	0	2	1	3
**037**	CC	ID	AT	GT	0	2	1	3
**038**	CT	DD	AA	GG	8	1	0	9
**039**	CC	ID	AA	GT	0	4	1	5
**040**	CT	II	TT	GT	2	0	2	4
**041**	CT	ID	AA	GG	1	3	8	12
**042**	CT	II	TT	GG	1	0	0	1
**043**	CC	DD	AA	GT	3	2	2	7
**044**	CC	DD	AT	GG	1	5	0	6
**045**	TT	DD	AT	GT	0	0	4	4
**046**	CT	ID	AT	GT	5	0	3	8
**047**	CT	ID	AT	GG	2	1	3	6
**048**	TT	DD	AA	GG	1	0	3	4
**049**	TT	DD	AT	GG	1	1	1	3
**050**	CC	ID	AA	GG	2	2	0	4
**051**	CT	DD	AT	GG	1	2	1	4
**052**	CC	DD	AT	GT	0	0	0	0
**053**	TT	ID	AT	GT	4	0	0	4
**054**	CT	ID	TT	GG	0	0	0	0
**055**	CT	II	AT	GT	1	2	1	4
**056**	CT	II	AT	TT	1	2	2	5
**057**	CC	II	TT	GG	0	2	0	2
**058**	CT	ID	AA	GT	0	0	5	5
**059**	TT	ID	AT	GG	2	2	0	4
**060**	TT	DD	AA	GG	0	0	0	0
**061**	CT	II	AA	GG	1	0	1	2
**062**	CC	DD	AT	GT	5	1	2	8
**063**	CT	II	TT	GG	3	4	3	10
**064**	CC	ID	AA	GG	3	3	3	9
**065**	CT	DD	AT	GG	0	1	0	1
**066**	CC	II	AA	GG	2	1	0	3
**067**	CC	ID	AT	GT	3	3	1	7
**068**	CT	ID	AT	GG	0	1	0	1
**069**	TT	ID	AA	GG	2	0	0	2
**070**	TT	DD	AT	GG	1	2	3	6

**071**	CT	ID	AA	GG	0	5	4	9
**072**	CT	II	AT	GT	1	2	2	5
**073**	TT	ID	AT	GG	0	0	1	1
**074**	CC	DD	AT	GT	1	1	0	2
**075**	CT	II	AT	GT	0	2	1	3
**076**	TT	ID	AT	GG	0	0	0	0
**077**	CC	ID	AT	GG	0	1	1	2
**078**	CC	DD	AA	GG	0	2	0	2
**079**	CC	ID	AT	GG	0	0	0	0
**080**	TT	ID	TT	GT	0	0	0	0
**081**	CT	ID	AA	GT	3	3	5	11
**082**	CT	ID	TT	GG	1	0	0	1
**083**	CT	II	AT	GT	1	0	0	1
**084**	TT	DD	AT	GT	0	0	2	2
**085**	CC	ID	AA	GT	0	1	1	2
**086**	CT	II	AA	GT	3	1	4	8
**087**	CT	DD	AT	GG	2	4	1	7
**088**	CC	DD	TT	GG	0	0	0	0
**089**	CT	DD	AT	GG	0	0	0	0
**090**	CT	ID	AT	GG	0	0	0	0
**091**	CC	DD	AA	GT	0	0	2	2
**092**	CT	DD	AT	GT	1	0	1	2
**093**	TT	ID	AA	GT	5	2	1	8
**094**	CC	ID	AT	GT	1	1	0	2
**095**	CT	ID	AT	GG	0	2	0	2
**096**	CC	DD	AT	GG	3	3	4	10
**097**	TT	ID	AT	GG	0	2	0	2
**098**	CT	ID	AT	GG	1	1	2	4
**099**	CT	ID	AT	GG	1	3	0	4
**100**	CC	DD	AT	GT	2	3	3	8
